# Supplementary material for: iNOS Gene Ablation Prevents Liver Fibrosis in Leptin-Deficient ob/ob Mice
Source: Genes (Basel). 2019 Feb 27;10(3):184. doi: 10.3390/genes10030184 (PMC6470935; doi:10.3390/genes10030184)
Supplement: Supplementary file 1 [file genes-10-00184-s001.zip › Genes 437083_Supplemental figure.pdf]

Supplemental Figure 1

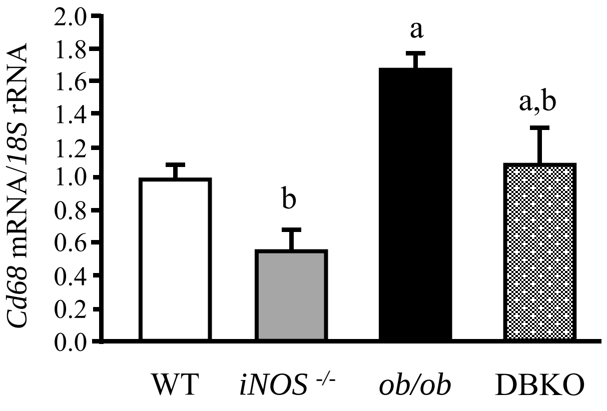

Supplemental Figure 1. Gene expression levels of *Cd68* in the liver. Leptin-deficient *ob/ob* mice exhibited a significantly increase in *Cd68* mRNA levels compared to wild type mice, with *iNOS* disruption reducing *Cd68* transcripts levels. Values are mean ± SEM (n=5 per group). The gene expression in WT mice was assumed to be 1. Differences between groups were analyzed by two-way ANOVA. [a]$P<0.05$ effect of the absence of the *ob* gene. [b]$P<0.05$ effect of the absence of the *iNOS* gene.

Supplemental Figure 2

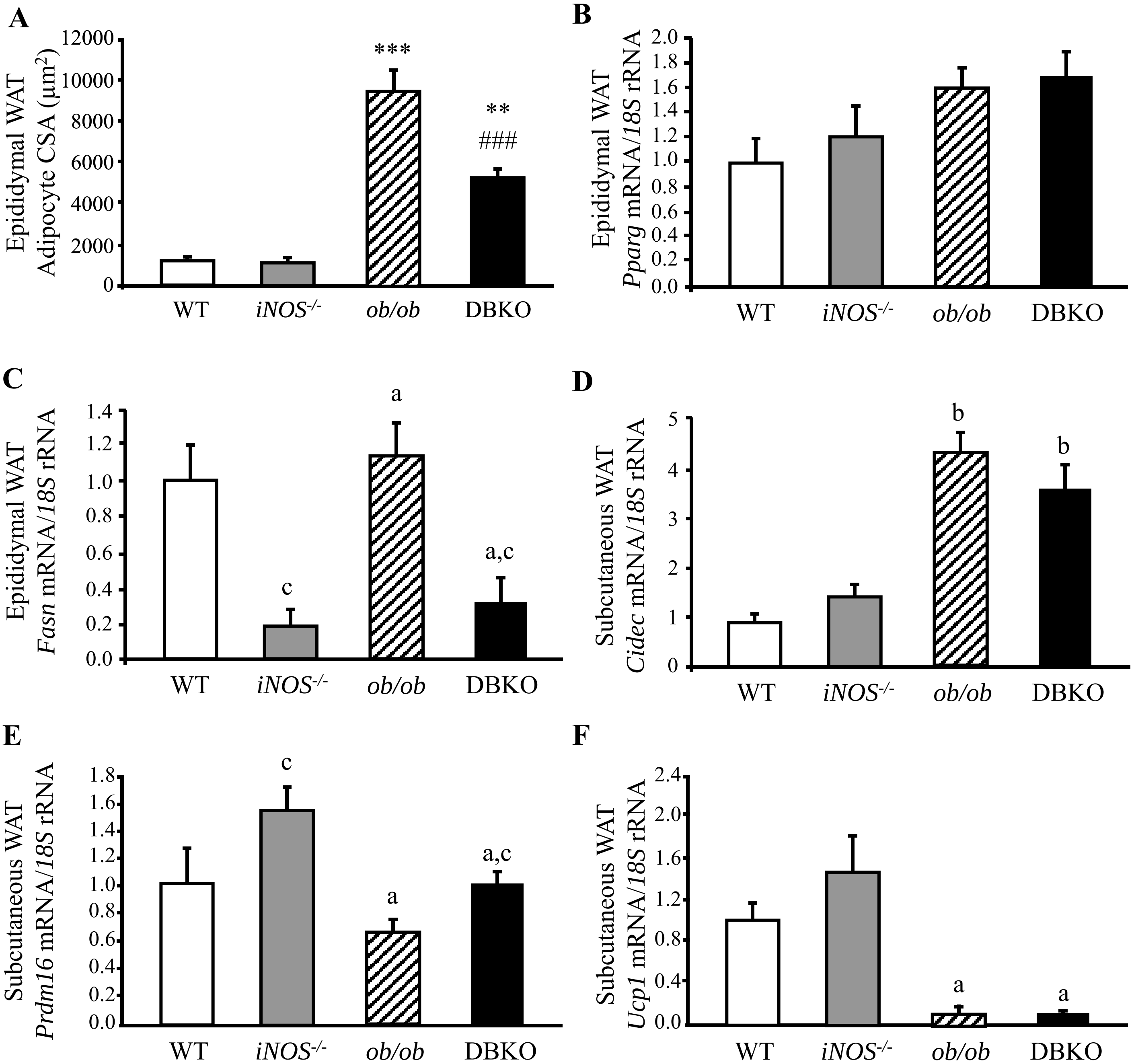

Supplemental Figure 2. Impact of iNOS deletion on markers of adipogenesis and fat browning in the context of leptin deficiency. (A) Adipocyte cell surface area in epididymal WAT (n=4-5 per group). Gene expression levels of markers of adipogenesis *Pparg* (B) and *Fasn* (C) in epididymal WAT and *Cidec* (D) in subcutaneous WAT as well as markers of fat browning *Prdm16* (E) and *Ucp1* (F) in subcutaneous WAT of experimental animals (n=6 per group). The gene expression levels in wild type was assumed to be 1. Differences between groups were analyzed by two-way ANOVA or one-way ANOVA followed by Tukey's *post hoc* test when an interaction between factors was detected. $^{a}p<0.05$, $^{b}p<0.001$ effect of the absence of *ob* gene, $^{c}p<0.01$ effect of the absence of the *iNOS* gene. ***$p<0.001$ *vs* WT mice; ###$p<0.01$ *vs* *ob/ob* mice.

Supplemental Figure 3

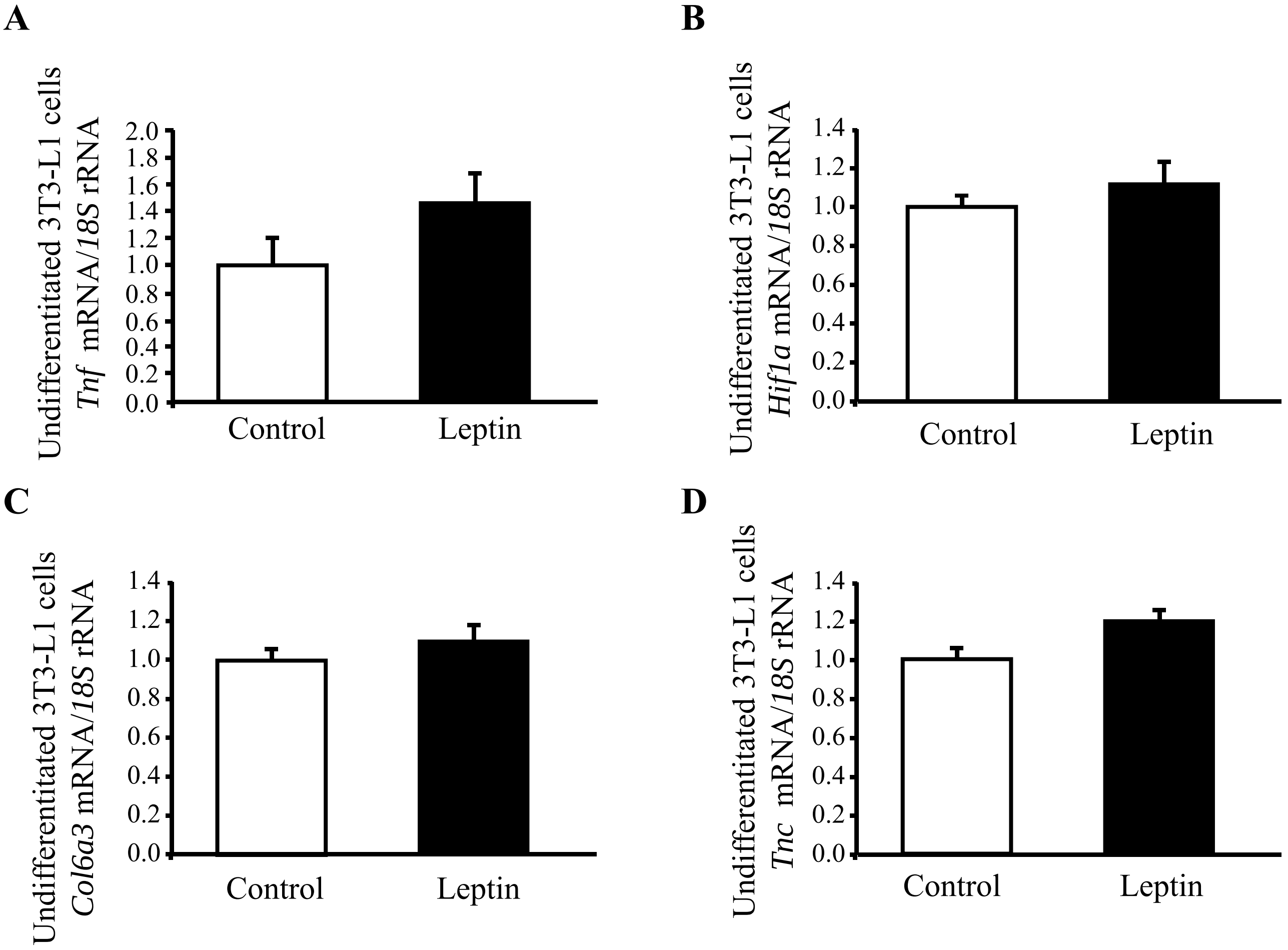

Supplemental Figure 3. Effect of leptin treatment on the expression of markers of inflammation and fibrosis in undifferentiated 3T3-L1 cells. mRNA expression levels of *Tnf* (A), *Hif1a* (B), *Col6a3* (C) and *Tnc* (D) in undifferentiated 3T3-L1 preadipocytes under basal conditions and after leptin (10 nmol L^{-1}) treatment for 24 h. The gene expression levels in unstimulated cells was assumed to be 1. Values are the mean ± SEM (n=6 per group). Differences between groups were analyzed by unpaired two-tailed Student's *t* tests.

Supplemental Figure 4

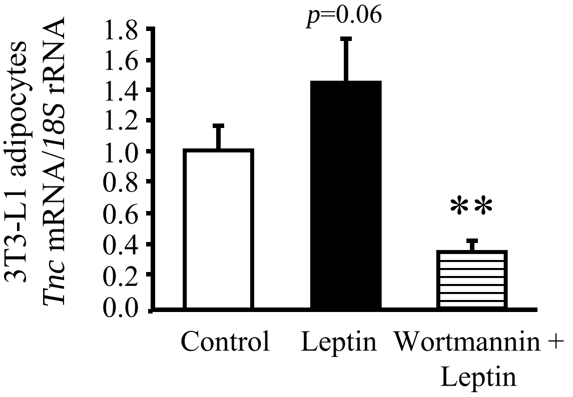

Supplemental Figure 4. Effect of leptin on *Tnc* gene expression in the presence of a PI3K inhibitor. *Tnc* transcript levels in 3T3-L1 adipocytes stimulated with leptin (10 nmol L^{-1}) in the absence or presence of PI3K inhibitor wortmannin (10 mmol L^{-1}) for 24 h. Gene expression levels in the unstimulated cells were assumed to be 1. Values are the mean ± SEM (n=5 per group). Differences between groups were analyzed by unpaired two-tailed Student's *t* tests . ***p*<0.01 *vs* unstimulated cells.
