# Supplementary material for: iNOS Gene Ablation Prevents Liver Fibrosis in Leptin-Deficient ob/ob Mice
Source: Genes (Basel). 2019 Feb 27;10(3):184. doi: 10.3390/genes10030184 (PMC6470935; doi:10.3390/genes10030184)
Supplement: Supplementary file 1 [file genes-10-00184-s001.zip › genes-437083-supplementary-tables.pdf]

Supplemental table 1. Sequences of the primers and TaqMan® probes.

**Gene** (GenBank accession number)	**Oligonucleotide sequence** (5’-3’)
Tnc (NM_011607)	
Forward	AAGAGCCAGCAAGCCACAAC
Reverse	ACAGCAGAAACACCAATCCCATA
TaqMan® Probe	FAM-ACCACACTCACAGGTCTAAGGCCCGG-TAMRA
Tnf (NM_013693)	
Forward	CCAGACCCTCACACTCAGATCAT
Reverse	ACTCCAGCTGCTCCTCCACTT
TaqMan® Probe	FAM-CCTGTAGCCCACGTCGTAGCAAACCA-TAMRA
Hif1a (NM_010431)	
Forward	TACCACACTGAGGTTGGTTACTGT
Reverse	AACTAGCCGAGGAAGAACTATGAACA
TaqMan® Probe	FAM- AACATGGAAGGTATTGCACTGCACAGGC-TAMRA
Col6a3 (NM_001243008)	
Forward	TGATGGCACCTCTCAGGACTCT
Reverse	TTGTCGGAGCCATCCAAAAG
TaqMan® Probe	FAM-CCACGGAAGTTCACGTAA-TAMRA
Col6a1 (NM_009933)	
Forward	CACCTGGGCCAGATGAGTGT
Reverse	CCAGCACGAAGAGGATGTCAA
TaqMan® Probe	FAM-AAATGTGCTCCTGCTGTGA-TAMRA
Col1a1 (NM_007742)	
Forward	TGTCCCAACCCCCAAAGAC
Reverse	GGTCCCTCGACTCCTACATCTTC
TaqMan® Probe	FAM-CTGCCCGGAAGAATACGTATCACCAAACTC-TAMRA
*Itgax (*NM_021334)	
Forward	CTGGACTTTGTTAAAGCTGTGATGAG
Reverse	GACGTGGAGATGAAGTTGTTGAAA
TaqMan® Probe	FAM-CCTAGCACACACGGTTCTCCCTGATGCA-TAMRA
Cd44 (NM_009851)	
Forward	AGAGCCGGAAGAAGACGAAAAC
Reverse	TCCACCTCTTCTTGCATCTTTAGC
TaqMan® Probe	FAM-CCACGGAAGTTCACGTAA-TAMRA

Emr1 (NM_010130)	
Forward	CAAGATTCTCTTCCTCACCGGTAT
Reverse	GCAGGCGAGGAAAAGATAGTGTAG
TaqMan® probe	FAM-CAACCAGACGGCTTGTGCCATCATT-TAMRA
Tlr4 (NM_021297)	
Forward	TGATGGCACCTCTCAGGACTCT
Reverse	TTGTCGGAGCCATCCAAAAG
TaqMan® Probe	FAM-CACCCAGCTGGCAGAGGCATACTTGT-TAMRA
Mmp9 (NM_013599)	
Forward	CCAAAGACCTGAAAACCTCCAA
Reverse	GCCCGGGTGTAACCATAGC
TaqMan® Probe	FAM-CCACCCAGCTGGCAGAGGCATACTTGT-TAMRA
Acta2 (NM_009338)	
Forward	GGCCACACGAAGCTGTTATAG
Reverse	GTATCCGATAGAACACGGCATCA
TaqMan® Probe	FAM-CATGGAAAAGATCTGGC-TAMRA
Spp1 (NM_009632)	
Forward	TTTGCCGTTTGGCATTGC
Reverse	TGGGTGCAGGCTGTAAAGCT
TaqMan® Probe	FAM- TCCTCCCTCCCGGTGAAAGT-TAMRA
Tgfb (NM_011480)	
Forward	TCCCAAGAGCCCTGCACTT
Reverse	TCCCAAGAGCCCTGCACTT
TaqMan® Probe	FAM- TTGACACGTTTCTTCCTGAGCAGCGC -TAMRA

Tnc: tenascin C*; Tnf*, tumor necrosis factor α; *Hifa*: hypoxia inducible factor 1,α subunit; *Col1a1*, collagen, type I, α1; *Col6a1*, collagen, type VI, α1; *Col6a3*, collagen, type VI, α3; *Itgax*, integrin α X (Cd11c); *Cd44*, Cd44 antigen; *Emr1*, egf-like module containing, mucin-like *Tlr4*: Toll like receptor 4; *Mmp9*, matrix metalloproteinase 9; *Acta2*, actin, α2; *Spp1*, secreted phosphoprotein 1; *Tgfb1*, transforming growth factor, β1.

Supplemental table 2. Metabolic characteristics of 12-week-old experimental animals.

	Wild type	*ob/ob* + PBS	*ob/ob* + leptin	*ob/ob* pair-fed
Body weight (g)	24.0 ± 0.4	44.9 ± 1.5***	24.8 ± 0.9$^{\dagger\dagger,\#\#}$	35.3 ± 1.0*
Liver weight (g)	1.04 ± 0.02	3.15 ± 0.12***	1.12 ± 0.08$^{\dagger\dagger,\#\#}$	1.30 ± 0.04**
FFA (mmol/L)	0.74 ± 0.08	1.07 ± 0.06***	0.77 ± 0.07$^{*,\#\#}$	1.60 ± 1.6*
TG (mg/dL)	66.3 ± 4.4	97.3 ± 5.3**	82.8 ± 11.0$^{**,\dagger,\#}$	147.9 ± 8.4
Glycerol (mg/dL)	0.025 ± 0.001	0.036 ±.0.001***	0.013± 0.003$^{**,\dagger\dagger\#\#}$	0.044 ± 0.006†,**
Glucose (mg/dL)	83 ± 5	410 ± 42***	175 ± 23**,††	169 ± 17††,**
Insulin (ng/mL)	0.42 ± 0.04	9.66 ± 0.61***	0.45 ± 0.08$^{*,\dagger\dagger,\#}$	2.58 ± 0.57†,*
HOMA	1.6 ± 0.2	172.3 ± 21.5***	3.81 ± 1.1$^{*,\dagger\dagger,\#}$	20.9 ± 5.8††
Adiponectin (mg/mL)	22.6 ± 4.1	16.6 ± 0.9***	39.5 ± 1.6*,††	37.0 ± 2.1*

FFA, free fatty acids; HOMA, homeostasis model assessment; TG, triacylglycerols. Data are mean ± SEM (n=4-5 per group). Differences between groups were analyzed by Kruskal-Wallis followed by Mann Whitney's *U* tests. $^{*}P$<0.05, $^{**}P$<0.01 *vs* wild type mice. $^{\dagger}P$<0.05, $^{\dagger\dagger}P$<0.01 *vs* vehicle-treated *ob/ob* mice. $^{\#}P$<0.05, $^{\#\#}P$<0.01 *vs* pair-fed *ob/ob* mice.
